# Is it just religious practice? Exploring patients’ reasons for choosing a faith-based primary health clinic over their local public sector primary health clinic

**DOI:** 10.4102/phcfm.v9i1.1219

**Published:** 2017-06-29

**Authors:** James D. Porter, Graham Bresick

**Affiliations:** 1 Madwaleni Hospital, Walter Sisulu University, South Africa; 2 School of Public Health & Family Medicine, Department of Family Medicine, University of Cape Town, South Africa

## Abstract

**Background:**

Person-centred, re-engineered primary health care (PHC) is a national and global priority. Faith-based health care is a significant provider of PHC in sub-Saharan Africa, but there is limited published data on the reasons for patient choice of faith-based health care, particularly in South Africa.

**Aim:**

The primary objective was to determine and explore the reasons for patient choice of a faith-based primary care clinic over their local public sector primary care clinic, and secondarily to determine to what extent these reasons were influenced by demography.

**Setting:**

The study was conducted at Jubilee Health Centre (JHC), a faith-based primary care clinic attached to Jubilee Community Church in Cape Town, South Africa.

**Methods:**

Focus groups, using the nominal group technique, were conducted with JHC patients and used to generate ranked reasons for attending the clinic. These were collated into the top 15 reasons and incorporated into a quantitative questionnaire which was administered to adult patients attending JHC.

**Results:**

A total of 164 patients were surveyed (a response rate of 92.4%) of which 68.3% were female and 57.9% from the Democratic Republic of the Congo (DRC). Of patients surveyed, 98.2% chose to attend JHC because ‘the staff treat me with respect’, 96.3% because ‘the staff are friendly’ and 96.3% because ‘the staff take time to listen to me’. The reason ‘it is a Christian clinic’ was chosen by 70.1% of patients. ‘The staff speak my home language’ was given as a reason by 61.1% of DRC patients and 37.1% of South African patients. ‘The clinic is close to me’ was chosen by 66.6% of Muslims and 40.8% of Christians.

**Conclusion:**

Patients chose to attend JHC (a faith-based primary care clinic) because of the quality of care received. They emphasised the staff–patient relationship and patient-centredness rather than the clinic’s religious practices (prayer with patients). These findings may be important in informing efforts to improve public sector primary care.

## Background

In 2009, the World Health Organization issued a World Health Report calling for the renewal of primary health care (PHC).^1^ The report highlighted the failure of countries to adhere to comprehensive PHC as per the Alma Ata declaration and called for four sets of PHC reforms, the second of which is particularly relevant to this research: to reorganise health services around ‘people’s needs and expectations, so as to make them more socially relevant and more responsive to the changing world’.^1^ This reform is further driven by the concept of person-centred care embedded in the World Health Organization in its 2007 Policy Framework.^2^ Person-centred health care is described as a health system which is designed around the needs of the stakeholders and in which individuals and communities are ‘served by and able to participate in trusted health systems that respond to their needs in humane and holistic ways’.^2^

In this context, all aspects that contribute to the provision of holistic, person-centred PHC are vital, including that of faith-based institutions. Acknowledging faith-based preferences of patients can be considered a part of practising person-centredness.^3,4^ In addition, there is an increasing realisation that ‘religious and spiritual concerns are important for understanding health-related behaviours’,^5^ which is particularly relevant in sub-Saharan Africa where 87% of the population identifies as either Muslim or Christian.^6^ Faith-based facilities account for a significant proportion of health care in many African countries, sometimes providing between 30% and 40% of hospital beds.^7,8^ They are active in the most rural of communities as well as in urban areas, and are ‘uniquely well placed to reach people to provide a range of services to those in need’.^9^ Despite this close alignment with community needs, faith-based organisations often go unrecognised because they tend to operate outside of government structures and planning processes.^10^

This realisation has led to an increased emphasis on research into faith-based health care, particularly in the developing world.^10,11,12^ Through efforts led by the World Health Organization, the World Bank and international collaborations such as the African Religious Health Assets Programme, sub-Saharan Africa has seen a growing body of research focusing on faith-based organisations and health care.^9,10,11,13,14,15,16^

There is a general perception in Africa, but little firm data, that faith-based health care facilities provide a better quality of care than state facilities.^17^ It is unclear whether this is because of their religious nature or other aspects of their function.^18^ However, recent studies have provided more definitive data in this area.^7,8,18,19^ Research from Ghana shows that even with higher costs, faith-based health facilities still have higher rates of satisfaction. This was mostly attributed to intangible elements such as courtesy, trust and patient-centredness.^18^ Another Ghanaian study showed quality of care and patient respect were reasons why patients chose faith-based care.^7^ This was a recurring theme in research from Burkina Faso where the main reasons for the choice of a faith-based health care facility were lower costs, the good staff–patient relationship and overall quality of care.^8^ Further research across 18 African countries showed that one of the main reasons for not choosing a particular clinic is a lack of respect shown to patients by staff.^20^

While the assumption is that the religious aspect of faith-based health care is one of the main reasons for patient choice, recent research does not back this up. Research from Ghana suggests that only 6.3% of Christians and 12.5% of Muslims say that they chose to attend a faith-based health facility because of its religious affiliation.^19^ Similarly in Burkina Faso, only 14.6% of patients mention religious affiliation as a reason for choosing a faith-based health facility.^8^

Research into faith-based health care in South Africa is vitally important, particularly given the context where 79.8% of the population identifies itself as being nominally Christian.^21^ Historically, Christian churches have made a significant contribution to health care in South Africa. Before and during apartheid, American and European missionary health services attempted to bridge the vast, institutionalised health care gap between Black and White South Africans.^22^ Many effective mission hospitals and clinics were established throughout the country to meet the needs of the marginalised.^23^

Very little research has been conducted on faith-based health care facilities in South Africa, and Cape Town specifically. This is partly because of the fact that South Africa was one of the African countries that integrated colonial era mission-based hospitals and public sector primary care and therefore no longer has an extensive network of faith-based health care facilities.^24^ Because of the Human Immunodeficiency Virus (HIV) epidemic and the South African government’s initial slow response to it, faith-based organisations became active again in terms of health care provision for HIV particularly.^25^

In summary, a reading of the literature suggests that despite this South African resurgence of faith-based care, there is still a lack of knowledge with regard to patients’ preference for faith-based facilities over state facilities when presented with the choice. A better understanding of the reason behind patient preference will help to improve primary care services. This is aligned with the Healthcare 2030 policy document of the Western Cape Government, where the primary vision is that of ‘access to person-centred quality care’.^26^ It further states that the aim of developing a person-centred service involves engaging with patients by ‘listening to their concerns, needs and perspectives’ and ‘treating them with dignity and respect’.^26^ Similarly, the South African Department of Health’s policy paper on the proposed National Health Insurance also makes reference to a re-engineered PHC model which will ‘take account of the local context and acceptability’ and which will be ‘tailored to respond to local needs’.^27^ There is clearly a national and provincial focus on patient-centred care.

Despite almost two decades of structural reform and a commitment to achieving the goals of PHC, a number of obstacles are preventing its full implementation in the South African context.^22^ This is seen at the first contact level, with overburdened, poorly run community health centres which have less than ideal reputations and public perceptions.^28,29^ Although free PHC has been implemented, access has remained a problem to urban and rural patients alike.^30,31^ This was confirmed by the National Health Care Facilities Baseline Audit of 2012 where PHC facilities scored poorly across the country, especially with regard to person-centred care.^32^ One of six priority areas assessed was ‘positive and caring attitudes’ for which the national average PHC facilities score was 25%.^32^

In response to the need for some of the above desired service features and the apparent lack of access to good quality, holistic health care, Jubilee Health Centre (JHC) was started in 2006.^33^ JHC is a faith-based primary health clinic attached to Jubilee Community Church (an independent, non-denominational Christian church based in Observatory, Cape Town). It offers ‘professional, confidential and affordable PHC to the needy within its sphere of influence’ including voluntary counselling and testing for HIV, and a pregnancy help centre. Every patient attending the clinic ‘is cared for by a team, given professional attention and then ministered to in prayer’.^33^ The permanent, salaried staff include one doctor, three professional nurses, one counsellor and one administrator. There are also two doctors, three physiotherapists, one counsellor and three intercessionists (volunteers who pray with patients) who volunteer on a full-time basis. Patients are charged R10.00 per consultation and a once-off fee of R5.00 for acute medication. Chronic medication is charged at cost price. Since 2006, the ‘sphere of influence’ referred to in the JHC mission statement has grown rapidly. The patient population has increased significantly: the clinic now sees approximately 350 patients per month, which is an increase of 160% from 2011,^33^ and on 27 September 2015 moved into larger, purpose-built facility on the Jubilee Community Church property.^34^ It has also seen a dramatic widening of its drainage area, as well as an increase in the number and diversity of patients choosing to attend JHC, prompting the decision to explore patients’ reasons for choosing to attend the clinic.

This study hopes to inform local public sector policy and practice by providing data with regard to users’ preference for JHC. The results will provide JHC with valuable information about patient needs and preferences. The management of JHC has committed to using the results to assist them in improving the quality of their health care. The study results will also assist the local public sector clinics with regard to patient preference and highlight specific areas which they could improve on, thereby enabling them to deliver more person-centred care. The study will also provide a starting point for collaboration between JHC and the surrounding public sector clinics to the benefit of their patients.

This study seeks to determine whether the reasons why the users of JHC chose this primary care service reflect their need and expectation of person-centred care.

The primary objective was to determine and explore the reasons for the patients’ choice to attend JHC over their local public sector primary health clinic. The secondary objective was to determine to what extent demography influences reasons for choosing JHC over their local public sector primary health clinic.

## Research methods and design

### Study design

This was a cross-sectional, descriptive study which made use of mixed methods. Three focus groups, based on the Nominal Group Technique (NGT),^35^ generated the content for a questionnaire. Correlational analysis was used, linking the results with the various demographic details of the patients.

### Setting

The study was conducted at JHC, a faith-based primary health clinic attached to Jubilee Community Church (an independent, non-denominational church), based in Observatory, Cape Town. This is a middle-to-lower income urban area, with a significant immigrant population. There is good access to public health care with a primary health clinic, a community health centre and an academic hospital all within 3 km.

### Study population and sampling strategy

JHC has roughly 5000 patients on file. They do not have firm demographic data available but estimate that 65% of their patients are from Francophone central Africa. The majority of these are illegal immigrants or refugees and asylum seekers. The next largest demographic includes local patients from the Woodstock, Salt River, Observatory area. The majority of these patients self-identify as ‘coloured’ and speak English or Afrikaans as their first language.

#### Phase 1: Focus groups

The three focus groups were formed using convenient and purposive sampling with an attempt to have an element of homogeneity in each group. The first focus group comprised all the available JHC staff. The second focus group consisted of patients from Francophone Africa and the third focus group comprised South African patients. The sampling for the patient focus groups was focused on finding key informants recommended by the staff members as well as recruiting volunteers by advertising the focus groups in the clinic waiting area.

Inclusion criteria for the focus groups were that patients needed to be 18 years old or above, have attended JHC at least three times before, have attended a public health facility at least once and be fluent in English, Afrikaans, Xhosa or French and be able to provide consent. Because of the nature of the NGT method, patients also needed to be literate.

#### Phase 2: Survey

A sample size of 163 was calculated for the survey using a sample calculator for a descriptive study of a continuous variable. This was based on a confidence interval of 95%, a margin of error of 5% and an expected proportion of 10.4%. The expected proportion was determined as the proportion of patients who would cite the religion aspects of the clinic as a reason for attending JHC. This was derived from combined data from the two papers which met the criteria for the literature review.^8,19^

Sampling for the survey was consecutive as the questionnaire was offered to every patient attending JHC across a three-week period in November 2014. Consecutive sampling was used because of the limited time available for data collection and the relatively small number of patients attending JHC.

The inclusion criteria for the survey included patients aged 18 years and above, able to consent and fluent in English, Afrikaans, Xhosa or French. Literacy was not an inclusion criterion as the questionnaire was interviewer administered.

### Data collection

#### Phase 1: Focus groups

The three focus groups were conducted using the NGT. They were conducted by the principal investigator and a trained assistant or translator. Each focus group was presented with the question ‘what are some of the reasons why you have chosen to attend Jubilee Health Centre?’ Individual participants were allowed to nominate as many answers as possible which were then collated and clarified. Each participant ranked his or her top five reasons of those collated, and these scores were combined to give a final group ranking.

#### Phase 2: Survey

The data generated by the focus groups were used to generate a questionnaire. The first section of the questionnaire was designed to obtain a variety of demographic data from the respondents. The second part of the questionnaire was informed by the focus group results. The top 10 ranked reasons from each focus group were combined to form 15 statements relating to why patients might choose to attend JHC. The respondents were then asked to agree or disagree with each statement by choosing an answer ranging from ‘strongly disagree’ to ‘strongly agree’ (including the option of ‘unsure’) based on a Likert scale for each of the 15 statements.

### Data analysis

The survey consisted of a range of demographic data and the Likert scale based responses to the various statements of why patients chose to attend JHC. Responses were recorded and analysed by using Microsoft Excel. The Likert scale responses were converted into binary variables and used to establish proportions. ‘Definitely’ and ‘maybe’ responses were combined and interpreted as a positive response while ‘maybe not’, ‘definitely not’ and ‘unsure’ responses were combined and interpreted as a negative response. Because of the small size of some of the demographic samples, a Fisher’s exact test was used to determine which demographic variables produced significantly different results for why patients chose to attend JHC.

### Ethical consideration

As this was a descriptive study without an intervention, there was very little potential for risk or discomfort. The only potential risk was covered in the establishment of a distress protocol, should a patient have found the survey or focus group to be distressing.

Informed consent was obtained for both the focus group and the survey by either the principal investigator or a research assistant and was offered in English, Afrikaans, Xhosa or French. The survey was offered to each patient only after their consultation with the doctor or nurse.

The study included immigrants even though they are considered to be a vulnerable population. It was considered essential to include them as they make up the majority of the clinic patient population, and there will be significant future benefits for this sub-group as a result of this research. Their confidentiality and privacy were emphasised to them. They did not have to disclose their legal status, and no identifying data such as date of birth or actual physical addresses were recorded. As for all patients, it was emphasised that whether or not they participated in the research would in no way affect the health care they received at JHC or any other facility.

This study was approved by the University of Cape Town’s Human Research Ethics Committee (HREC REF: 118/2014).

## Results

### Phase 1: Focus groups

Six participants were recruited for the JHC staff focus group, 10 in the Francophone African focus group and 9 in the South African focus group. The staff focus group produced 28 responses to the question ‘what are some of the reasons why you have chosen to attend Jubilee Health Centre?’ The Francophone African focus group resulted in 19 responses and the South African focus group provided 36 responses. Table 1 shows the top 10 ranked answers from each focus group. These 30 reasons for choosing to attend JHC were collated to provide 15 statements which were used in the survey (see Table 3) to be agreed or disagreed with using a Likert scale.

**TABLE 1:** Top 10 focus group reasons for choosing to attend Jubilee Health Centre.

Rank†	Francophone African focus group	South African focus group	JHC staff focus group
1	Staff take good care of me	The clinic is affordable	Patients’ home languages are spoken
2	It is easier to see a doctor	Staff pray with me	The clinic is cheap
3	It is a Christian clinic	Staff have godly wisdom	Patients feel respected
4	Staff take time to listen to me	The clinic is clean and neat	Staff are friendly
5	Staff pray with me	Staff treat me with respect	Patients feel heard and listened to
6	The clinic is cheap	Staff are good listeners	Consultations are thorough
7	Staff give good medication	Often see the same doctor	Patients feel taken care of
8	Staff are friendly	Staff are good with children	Patients are treated with dignity
9	I trust the staff to be confidential	Staff have patience with me	Patients feel loved
10	The clinic is close to me	Staff are confidential	Recommended by word-of-mouth

JHC, Jubilee Health Centre.

† Ranked by focus group participants according to NGT process.

### Phase 2: Survey

A total of 185 patients were invited to participate in the survey. Of those, 14 declined to participate and 7 were excluded based on their inability to communicate effectively in English, Afrikaans, Xhosa or French. This left 164 participants who completed the questionnaire.

The patients surveyed were predominantly female, married and between the ages of 31 and 45 years. Only 21.3% of the patients were from the Republic of South Africa (RSA) with the majority (57.9%) from the Democratic Republic of the Congo (DRC). Accordingly, French, Lingala and Swahili were the most commonly spoken home languages. Those identifying with the Christian faith made up 86.6% of the patients (only 12.8% attending Jubilee Community Church) with a small but significant number of Muslim patients (12.8%). The average socioeconomic status is shown by the fact that 56.1% of patients were unemployed with the average, monthly household income between R1000 and R5000. The vast majority (82.9%) had attended a public sector clinic before, with Spencer Road Clinic (in Observatory) the most common closest public sector clinic. However, only 54.3% of patients were able to correctly name their closest public sector clinic. Based on residential suburbs (actual physical addresses were not recorded), it was calculated that on average, unemployed patients travel just over five times further to get to JHC (9.7 km) than would be needed to get to their closest public sector clinic (1.8 km), with 34.2 km the furthest distance travelled to get to JHC by an unemployed patient.

**TABLE 2:** Demographics of survey respondents (*n* = 164).

Category	*n*	%
**Age**		
18–30 years old	48	29.3
31–45 years old	70	42.7
46–60 years old	35	21.3
> 60 years old	11	6.7
**Gender**		
Female	112	68.3
Male	52	31.7
**Relationship status**		
Married	116	70.7
Single	28	17.1
Other	20	12.1
**Home language**		
French	41	25.0
Lingala	33	20.1
Swahili	17	10.4
Afrikaans	16	9.8
English	16	9.8
Other	41	25.0
**Home country**		
DRC	95	57.9
RSA	35	21.3
Zimbabwe	8	4.9
Malawi	7	4.3
Burundi	6	3.7
Other	13	7.9
**Religion**		
Christian	143	87.2
Muslim	21	12.8
**Monthly household income**		
< R1000	57	34.8
R1000–R5000	93	56.7
> R5000	14	8.5
**Employment status**		
Unemployed	92	56.1
Employed	50	30.5
Self-employed	22	13.4
**Attended public sector clinic before**	136	82.9
**Jubilee Community Church member**	21	12.8
**Closest public sector clinic**		
Spencer Road Clinic	48	29.3
Woodstock CHC	16	9.8
Lady Michaelis CHC	10	6.1
Goodwood Clinic	10	6.1
Other	80	43.3
Correctly identified closest clinic	89	54.3

*n*, number; DRC, Democratic Republic of the Congo; RSA, Republic of South Africa.

The second half of the questionnaire provided the results to the Likert scale responses to the various reasons for choosing to attend JHC. The responses were binarised by combining ‘definitely’ and ‘maybe’. As seen in Table 3, the top five reasons were separated by only 3.7% (98.2% to 94.5%). ‘The staff treat me with respect’ was the highest ranked answer, followed by ‘The staff are friendly’, ‘The staff take time to listen to me’, ‘It is easier to see a doctor’ and ‘The staff give the correct treatment for my illness’. Reasons related to the religious aspects of the clinic (‘It is a Christian clinic’ and ‘The staff pray with me’) rank seventh and eighth out of the 15 reasons (70.1% and 61%). The cost of clinic was the least chosen reason for attending JHC with 39%.

**TABLE 3:** Survey reasons for choosing to attend Jubilee Health Centre (*n* = 164).

Rank†	Likert scale statements	*n*‡	(%)
1	The staff treat me with respect	161	98.2
2	The staff are friendly	158	96.3
2	The staff take time to listen to me	158	96.3
4	It is easier to see a doctor	155	94.5
4	The staff give the correct treatment for my illness	155	94.5
6	It was recommended to me by others	124	75.6
7	I can trust the staff to be confidential	122	74.4
8	It is a Christian clinic	115	70.1
9	The staff pray with me	100	61.0
10	The staff treat children well	94	57.3
11	I often am seen by the same doctor	86	52.4
12	The clinic is clean and neat	81	49.4
12	They speak my home language	81	49.4
14	It is close to me	73	44.5
15	It is cheap	64	39.0

*n*, number.

† Out of 15 statements.

‡ Binarised by combining ‘definitely’ and ‘maybe’ responses

The reasons for choosing to attend JHC were further broken down with various demographics as shown in Table 4a-c. Patients from the DRC were compared with those from the RSA, and three reasons for attending JHC were found to have statistically significant differences. Almost two-thirds of Congolese patients said that they attended JHC because their home language was spoken as opposed to 37.1% of South African patients. Close to 100% of Congolese patients attended JHC because it was easier to see a doctor compared to 88.6% of South Africans. Roughly a quarter of patients from the RSA chose to attend JHC because their children were treated well, compared to about two-thirds of patients from the DRC.

**TABLE 4a:** Comparison between demographic groups of reasons for choosing to attend Jubilee Health Centre.

Survey reasons for choosing to attend JHC	DRC (*n* = 95)			RSA (*n* = 35)			*p*
	*n*	%	Rank	*n*	%	Rank	
The staff treat me with respect	95	100.0	1	34	97.1	1	0.2692
It is easier to see a doctor*	94	98.9	2	31	88.6	5	0.0185
The staff take time to listen to me	93	97.9	3	33	94.3	3	0.5735
The staff are friendly	91	95.8	4	34	97.1	1	1.0000
The staff give the correct treatment for my illness	90	94.7	5	33	94.3	3	1.0000
I can trust the staff to be confidential	74	77.9	6	26	74.3	6	0.8148
The clinic was recommended to me by others	69	72.6	7	26	74.3	6	1.0000
It is a Christian clinic	66	69.5	8	23	65.7	9	0.8318
The staff speak my home language*	58	61.1	9	13	37.1	12	0.0179
The staff treat children well**	56	58.9	10	9	25.7	15	0.0014
The clinic is clean and neat	53	55.8	11	13	37.1	12	0.0755
The staff pray with me	53	55.8	11	26	74.3	6	0.0690
The clinic is cheap	43	45.3	13	11	31.4	14	0.1672
I often am seen by the same doctor	43	45.3	13	22	62.9	10	0.1130
The clinic is close to me	39	41.1	15	15	42.9	11	1.0000

JHC, Jubilee Health Centre; *n*, number; DRC, Democratic Republic of the Congo; RSA, Republic of South Africa.

* *p* < 0.05 (Fisher’s exact test).

** *p* < 0.01 (Fisher’s exact test).

**TABLE 4b:** Comparison between demographic groups of reasons for choosing to attend Jubilee Health Centre.

Survey reasons for choosing to attend JHC	Christian (*n* = 142)			Muslim (*n* = 21)			*p*
	*n*	%	Rank	*n*	%	Rank	
The staff treat me with respect	139	97.9	1	21	100.0	1	1.0000
The staff are friendly	136	95.8	2	21	100.0	1	0.6035
The staff take time to listen to me	136	95.8	2	21	100.0	1	0.6035
It is easier to see a doctor	135	95.1	4	19	90.5	4	0.6059
The staff give the correct treatment for my illness	135	95.1	4	19	90.5	4	0.6060
It is a Christian clinic**	113	79.6	6	2	9.5	15	0.0000
I can trust the staff to be confidential	109	76.8	7	12	57.1	9	0.0649
The clinic was recommended to me by others	105	73.9	8	18	85.7	6	0.2905
The staff pray with me**	95	66.9	9	4	19.0	12	0.0000
The staff treat children well	81	57.0	10	13	61.9	8	0.8141
The clinic is clean and neat*	76	53.5	11	4	19.0	12	0.0042
I often am seen by the same doctor	75	52.8	12	10	47.6	10	0.8156
The staff speak my home language	71	50.0	13	9	42.9	11	0.6423
The clinic is cheap	60	42.3	14	4	19.0	12	0.0548
The clinic is close to me*	58	40.8	15	14	66.7	7	0.0339

JHC, Jubilee Health Centre; *n*, number.

* *p* < 0.05 (Fisher’s exact test).

** *p* < 0.01 (Fisher’s exact test).

**TABLE 4c:** Comparison between demographic groups of reasons for choosing to attend Jubilee Health Centre.

Survey reasons for choosing to attend JHC	Employed (*n* = 50)			Unemployed (*n* = 92)			*p*
	*n*	%	Rank	*n*	%	Rank	
The staff take time to listen to me	50	100.0	1	87	94.6	3	0.3108
The staff treat me with respect	50	100.0	1	89	96.7	1	0.1618
The staff are friendly	48	96.0	2	89	96.7	1	1.0000
It is easier to see a doctor	48	96.0	2	86	93.5	4	0.7126
The staff give the correct treatment for my illness	48	96.0	2	86	93.5	4	0.7126
I can trust the staff to be confidential*	42	84.0	6	63	68.5	7	0.0478
The clinic was recommended to me by others	42	84.0	6	68	73.9	6	0.2094
It is a Christian clinic	33	66.0	8	63	68.5	7	0.8515
The staff pray with me	33	66.0	9	54	58.7	9	0.4716
The staff treat children well	31	62.0	10	48	52.2	10	0.2919
The clinic is clean and neat	30	60.0	11	39	42.4	14	0.0540
I often am seen by the same doctor	28	56.0	12	46	50.0	11	0.5981
The clinic is cheap**	27	54.0	13	25	27.2	5	0.0020
The clinic is close to me	27	54.0	13	40	43.5	13	0.2912
The staff speak my home language	25	50.0	15	41	44.6	12	0.5986

JHC, Jubilee Health Centre; *n*, number.

* *p* < 0.05 (Fisher’s exact test).

** *p* < 0.01 (Fisher’s exact test).

When comparing patients who identified with Christianity as opposed to patients who identified with Islam, four statistically significant differences (*p*-value < 0.05 using the Fisher’s exact test) were found in response to why patients chose to attend JHC. One-fifth of Muslim patients chose to come to JHC because ‘The clinic is clean and neat’ compared to just over half of those identifying with Christianity. The religious aspects of the clinic also recorded significant differences with 9.5% of Muslims choosing to attend JHC because it is a Christian clinic and 19% choosing to attend because they were prayed with. In comparison, almost 80% of Christians chose to attend JHC because of its religious affiliation and 66.9% agreed with the statement ‘I chose to come to Jubilee Health Centre because the staff pray with me’. The final significantly different response was to the statement ‘I chose to come to Jubilee Health Centre because it is close to me’ with 66.6% of Muslims and 40.8% of Christians agreeing with it.

Those patients who were formally employed were also compared to those who were unemployed. Two statistically different responses were found. Of those patients formally employed, 84% said they came to JHC because they could trust the staff to be confidential compared to 68.5% of those who were unemployed. Just over half of the employed patients chose to come to JHC because it is cheap compared to just over a quarter of unemployed patients.

## Discussion

The primary objective of this study was to determine and explore the reasons for patients’ choice to attend JHC (a faith-based primary health clinic) over their local public sector primary health clinic. Although the patient focus groups gave high rankings to the religious aspects of the clinic (third and fifth in the Francophone African focus group and second and third in the South African focus group), the survey showed that the top three reasons for choosing to attend JHC were instead related to the quality of the care received, specifically with reference to the staff–patient relationship and the respect and attention paid to patients. The actual religious aspects of the clinic were not the predominant reasons for choosing the clinic, ranking eighth and ninth out of the top 15 reasons.

The secondary objective was to determine to what extent sociodemographic factors determine the reasons for choosing JHC. The significant demographic results were that almost 60% of the patients were from the DRC with South Africans comprising the next biggest population group (21.3%). Although patients from the DRC placed equal emphasis on quality of care, they also ranked the treatment of children, home language spoken and ease in seeing a doctor higher than the South African patients. Despite the availability of a French, Lingala and Swahili translator and the higher ranking of ‘the staff speak my home language’ (*p* = 0.0179), patients from the DRC still only ranked it ninth out of 15 reasons. Although it did not achieve statistical significance (*p* = 0.0690), it is interesting to note that more South African patients chose to attend JHC because ‘the staff pray with me’ than patients from the DRC (74.3% compared to 55.8%). Other statistically significant differences were that Muslim patients ranked faith-based reasons in the bottom two compared to Christians who ranked them sixth and ninth and employed patients ranked ‘the clinic is cheap’ higher than unemployed patients.

The results suggest that the reasons for the patients’ choice to attend JHC are not related to religion but rather to the quality of the care provided. Respect, attention paid to patients and the friendliness of staff were particularly emphasised. Although there is a paucity of literature, these findings are in keeping with two previous studies from Ghana and Burkina Faso.^8,19^ Shojo et al. also found that the reasons patients chose faith-based care were ‘not related to religion per se, but rather to the quality of the services provided, including (but not only) through the values of dignity and respect for patients’.^19^ Gemignani et al. found that patients felt the quality of services were higher at faith-based facilities than at public facilities because of the ‘ways of speaking to patients, the ability to work within the local cultural context, and attention not just to disease but to a patient’s sense of wellbeing’.^8^

A potential divergence from the literature was the response rate to statements concerning the faith elements of the clinic. Shojo et al. found that only 6.3% of patients making use of Christian clinics mentioned religious aspects as reasons for choosing to attend,^19^ while Gemignani et al. found that in Burkina Faso, 14.6% of patients mentioned religious affiliation as a reason for choosing faith-based care.^8^ In contrast, this study found that although ranked eighth and ninth, 70.1% of patients chose JHC because it is a Christian clinic and 61.0% chose it because of the prayer offered by staff members. When considering the South African patient sub-group, 74.3% chose to attend JHC because the staff prayed with them.

### Limitations

The difference in response rates for religious factors when compared to the literature might be explained by the fact that this study was conducted on-site, a factor which is known to result in more favourable responses. Another possible explanation might be that the element of choice (compared to other countries where local public sector primary care clinics might not be as accessible) could have introduced a self-selection bias in favour of the religious features of JHC.

The difference in rankings between the patient focus groups and the results of the survey might be explained by focus group selection bias. Focus group participants were mostly considered key informants and either volunteered or were recommended by the JHC staff. This might have resulted in an over-sampling of Jubilee Community Church members or JHC advocates.

A possible reason for the overall higher proportions of the responses in the survey may be the use of Likert scale responses to determine reasons for choosing JHC, that is, patients were limited to a pre-determined range of options when responding to each of the 15 statements. Volunteering their own reasons (as in semi-structured interviews) may have yielded a different result. This may have resulted in the over-representation or over-reporting of various reasons but will hopefully not have affected the rankings or comparative proportions of the 15 reasons for choosing JHC.

The relatively small sample size of 164, especially when it comes to sub-group analysis by demographic variables (only 21 Muslim patients were surveyed), is also a limitation. The sample size calculation was based on the total number of respondents and not stratified by sub-group. This necessitated the use of Fisher’s exact tests when comparing sub-groups.

### Recommendations and implications

Despite these limitations, recommendations can be made from this study. Its increasing attendance figures and the distances patients are willing to travel to attend the clinic (up to a 68.4 km round trip) suggest an improvement in JHC’s services compared to public sector clinics in the area. The implication of the results from the survey is that the quality of care provided by JHC is of a high standard and is attracting patients to the clinic. This reflects the need and expectation of patients for person-centred care increasingly referred to in current literature and policy statements.^2,36^ The Western Cape Government, as detailed in its Healthcare 2030 policy document, is attempting to improve collaboration between non-profit and community-based organisations which ‘have become increasingly important as providers of community-based services’.^26^ This study provides a starting point for collaboration between JHC and the surrounding public sector clinics to the benefit of their patients. Future research should be directly comparative between public sector and faith-based health care to more accurately depict strengths and weaknesses. Exploring comparative satisfaction rates between JHC and its closest public sector clinics would help to highlight the differences and where public sector clinics (and JHC) can improve.

Further investigation is needed to determine what the link is between increased quality of care and faith-based health providers. A Ugandan study found that staff in faith-based facilities had higher performance than those in public facilities, attributed to their motivation by the ‘faith-based organisational ethos’.^37^ Schmid et al. make mention of the impact on work ethic and quality of care by the religious commitment of health workers in Uganda, Zambia and Mali.^15^ Olivier et al., in the recent faith-based health care series in the Lancet, state that ‘the connection between faith-based values and health systems performance needs substantially more attention to be able to inform policy-level action’.^12^

This study also implied that faith-based aspects of care might be more important to South Africans than suggested in the literature,^8,19^ as 74.3% of them chose to attend JHC because of the prayer offered. This warrants further research into the area of faith-based health care and patient preferences in a South African context and even a local Cape Town context. Olivier et al. recommend that ‘we need to move away from broad generalisations of the magnitude and character of faith-based organisations and instead find out how different kinds of faith-based health providers operate within different contexts and systems’.^12^

## Conclusion

This study found that although there was a higher response rate than in the literature, the religious features of JHC (its Christian identity and the prayer offered with every consultation) were not the main reasons that patients chose to attend the clinic. Rather, the results show that the quality of the care received, with emphasis on the staff–patient relationship and patient-centredness, determined patients’ reasons for choosing JHC.

When exploring the demographic factors of the clinic population, it was found that almost two-thirds of patients were from the DRC. Although more Congolese patients chose to attend the clinic because they were addressed in their home language when compared to South Africans, their top reasons for choosing JHC were still those relating to quality of care.

This emphasis on quality of care is in alignment with the provincial, national and global focus on re-engineered, person-centred PHC. Further research into the role and importance of faith-based health care in South Africa, differences in satisfaction rates between public sector and faith-based health care, and possible avenues for collaboration, is recommended.
